# Health-related quality of life in adolescents with chronic physical illness in northern Russia: a cross-sectional study

**DOI:** 10.1186/1477-7525-12-12

**Published:** 2014-01-24

**Authors:** Anna Zashikhina, Bruno Hagglof

**Affiliations:** 1 Division of Child and Adolescent Psychiatry, Department of Clinical Science, Umea University, Umeå S-901 87, Sweden

**Keywords:** Adolescents, Chronic physical illness, Disease severity, Gender, Health-related quality of life

## Abstract

**Background:**

Health related quality of life (HRQoL) is an important subjectively evaluated outcome of adolescents physical, mental, and social functioning. It gives us the possibility to assess the disease impact on life of adolescents, and to sort out target groups of adolescents for future psychological interventions. The objective of this cross-sectional survey was to study HRQoL in 173 adolescents with chronic physical illness (CPI - diabetes, asthma, and epilepsy), and to find HRQoL predictors in each disease group.

**Methods:**

Disease-specific questionnaires were completed by each adolescent recruited from the local outpatient clinic; mothers answered the questions on socioeconomic status (SES); and the patients’ clinicians evaluated the severity of the disease.

**Results:**

A high proportion of adolescents in each disease specific sample reported moderate to high levels of HRQoL. Gender was the most prominent predictor of HRQoL in all three studied groups, while disease severity predicted HRQoL in the diabetic group and to some extent in the asthma group.

**Conclusions:**

Our results provide evidence that adolescents with diabetes, asthma, and epilepsy in northern Russia maintain relatively moderate to high levels of HRQoL. The domains affecting HRQoL were related to both disease-specific (severity) and non-disease factors (gender and SES). Our study suggests that future psychosocial interventions should focus on aspects of CPI impacting adolescents in gendered ways, furthermore taking into account disease specific factors.

## Background

Health-related quality of life (HRQoL) is an important outcome in studies of paediatric patients with chronic physical illnesses (CPI) such as asthma, diabetes or epilepsy [1-3]. Research has shown that HRQoL in children and adolescents with CPI is markedly lower than that of the general population [3,4]. HRQoL is generally considered as a multi-factorial construct focusing on individuals’ subjective evaluations of their physical health, mental health and social functioning [4] and the objective impact of the disorder associated with the disease or medical treatment [5]. Disease management and treatment routines often disrupt a child’s usual activities, requiring compliance with health regimes and placing extensive behaviour demands on children, which could potentially impact HRQoL. There is evidence that the burden of chronic illness has been growing for adolescents [6]. Developing any CPI during puberty could be very challenging; similarly for those who had already had a CPI for some time, as entering adolescence might mean finally facing up to the demands that the condition entails. “Fitting in” with one’s peer group is an important component of socialization, while self-discipline and disease compliance may become viewed as distinguishing and stigmatizing features of the illness, differentiating the CPI adolescent from his or her peers. This might, therefore, have a deleterious effect on HRQoL. Zullig and colleagues [7] documented the relationship between poor physical health and life dissatisfaction (as a subjective component of quality of life (QoL)) in an adolescent population, which gives us reason to assume that HRQoL in paediatric patients might mostly depend on disease severity. By disease severity, we mean physiological severity reflecting interactions of biological severity with environmental factors including medical treatment [8]; for instance, in a study by Stevanovich [9] medication intake was the most significant predictor of HRQoL in adolescents with well-controlled epilepsy. Graue et al. showed the complexity of the variables relating to the perception of HRQoL in adolescents with type 1 diabetes. Their results stated that HRQoL is not primarily determined by the daily burden of the disease compliance and clinical factors, but mostly by age and gender of the participants [10]. Alvim et al. reported that HRQoL of adolescents with asthma is influenced by the complex interaction among: the severity of clinical symptoms, morbidity, gender and the psychological resources available [11]. Therefore, the non-disease specific factors might play a determinative role in self-perceived HRQoL. Gender has been shown to affect HRQoL perception in adolescents. Girls tend to rate their QoL lower than boys, expressing more disease-related anxieties and less life satisfaction [11,12]. Others suggest that chronic condition threatening dominant masculinity may put boys into a vulnerable state [13]. Age was also confirmed as a strong predictor of CPI HRQoL. In most studies, younger children reported greater impairment [11,14,15].

These connections have not been thoroughly explored in adolescents with CPI in northern Russia. HRQoL in children in Russian Federation is an important and interesting topic to study with regard to the fall of the Soviet Union, the turmoil of the transition period during which our participants were born and raised. We assumed that the subjective evaluation of various functioning categories of CPI adolescents demonstrate not only the level of the healthcare system, but a well-being of the society. The health status of children is in the best interests of society as a whole. Our study focused on adolescents with diabetes, asthma or epilepsy, since these conditions are common in childhood [16]. Assessment of HRQoL in this sample could help us to describe and understand factors comprising self-perception of QoL in different disease groups. This will give us an opportunity to develop interventions based not only on improved medical care, but also on psychological and social adaptation of the adolescent patients. The aims of the study were to examine health-related quality of life in adolescents with CPI; and to investigate the impact of disease severity, disease duration, socioeconomic status, family type, age and gender on HRQoL.

## Methods

### Subjects

A total of 147 adolescents aged 13-16 years with type 1 diabetes (n = 50), asthma (n = 49) or epilepsy (n = 47), and their parents, were recruited from child outpatient clinics in Arkhangelsk, northern Russia. The adolescent’s CPI had to be diagnosed at least one year prior to data collection, as we were interested in long-term HRQoL rather than the stressful post-diagnosis adjustment period. Potential participants were excluded if they had mental retardation, lived in an institution, or had more than one chronic condition. Sociodemographic data for the study sample are given in Table 1.

**Table 1:** Some socio-demographic data (percentage within brackets) and disease severity status

**CPI n = 146**	**Diabetes**	**Asthma**	**Epilepsy**
Gender			
Male/Female n (%)	28(56)/22(44)	26(53)/23(47)	19(40.4)/28(59.6)
Mean age	14.15	14.25	14.95
Family SES n (%)			
Low	9 (18)	12 (24.5)	15 (31.9)
Average	28 (56)	17 (34.7)	17 (36.2)
High	12 (24)	16 (32.7)	14 (29.8)
Missing	1 (2.0)	4 (8.2)	1 (2.1)
Disease severity n (%)			
Non-severe CPI	29 (58)	24 (49)	34 (72.3)
Severe CPI	21 (42)	25 (51)	13 (27.7)

### Procedure

A total of 173 potential participants (90 girls and 83 boys) were identified from outpatient clinic records. All adolescents with diabetes in Arkhangelsk who were attending the paediatric outpatient clinic at the start of the data collection period (autumn 2002) and who met our inclusion criteria were asked to participate. Altogether, 55 adolescents with type 1 diabetes matched our inclusion criteria. For the purpose of our investigation, we decided to include approximately the same numbers of adolescents with asthma and epilepsy. Studying the outpatient clinic registers from the three largest districts of Arkhangelsk, we chose for recruitment every second adolescent with epilepsy (n = 59) and every third with asthma (n = 59) matching our criteria. Mothers of adolescents with CPI were contacted and their written informed consent was received, 91% of them agreed to participate in the study. All adolescents whose mothers gave consent assented prior to participating in the study. The refusal rates were: 7% in diabetes group, 8% in group with asthma, and the highest in the epilepsy group (15%). Five adolescents who were inpatients during the data collection period and two adolescents who did not complete the questionnaire were excluded from the study sample. Three families (two with adolescents with epilepsy and one with asthma) were unreachable. Adolescent participants completed, by hand, an established self-report questionnaires assessing HRQoL (described below). Questionnaires were completed either in the outpatient clinic or at home. In both settings, the investigator observed the completion of the questionnaire to minimize the bias of parent/child presence.

### Instruments

Based on the objectives of our study we decided to choose disease-specific rather than generic instruments. We were more interested in issues relevant to the target population.

#### ***The diabetes quality of life questionnaire for youths (DQOLY)***

The DQOLY, developed by the Diabetes Control and Complication Trial Research Group [17] and later modified by Ingersoll and Marrero [18], is composed of 52 items in four subscales: a Diabetes Life Satisfaction scale, a Diabetes Impact scale, a Diabetes-Related Worries scale, and a single item general self-rating of overall health in a response format of a four- or five-point Likert scale. In all subscales, except for the Life Satisfaction subscale, lower score indicated higher QoL. We recoded all scales in the same direction for ease of cross-scale comparison. The Hvidøre Study Group of Childhood Diabetes translated and used DQOLY in many countries. The questionnaire proved to be a reliable source of information on adolescent QoL, and a comprehensive and workable tool [19].

#### ***Paediatric asthma quality of life questionnaire (PAQLQ)***

The PAQLQ is 28-item questionnaire designed for children and adolescents with asthma [20,21]. It was derived from a QoL questionnaire for adults with asthma [22]. The child version uses a seven-point Likert scale that can be completed reliably by children as young as seven years. The PAQLQ includes a total score and three primary domains: how asthma interferes with Activities, asthma Symptoms, and Emotional reactions to asthma. Higher scores in these domains indicate better QoL. PAQLQ has been shown to have good measurement properties and high validity in both evaluative and discriminative studies [20]. Translation into Russian and linguistic validation of PAQLQ was made by the MAPI Research Institute in Lyon, France [23].

#### ***Quality of life in epilepsy inventory for adolescents (QOLIE-AD-48)***

This instrument has been developed and validated to assess multiple aspects of health-related quality of life (HRQoL) in adolescents with epilepsy [24]. The QOLIE-AD-48 contains 48 items in eight subscales: Epilepsy Impact (12 items), Memory/Concentration (10 items), Attitude toward Epilepsy (4 items), Physical Functioning (5 items), Stigma (6 items), Social Support (4 items), School Behaviour (4 items), Health Perception (3 items), and a Total score. The raw numbers of the five-point Likert scale for QOLIE-AD-48 domain scores were converted into a 0-100 point response scale, with higher scores indicating better HRQoL. QOLIE-AD-48 has been shown to be a specific, sensitive and reliable measure and has been translated and validated for Chinese, Serbian and Brazilian populations [25-27]. The developer (Ms. Joyce Cramer) provided the original QOLIE-AD-48 version. The translation of QOLIE-AD-48 and DQOLY into Russian followed established guidelines, including appropriate use of independent back translation [28].

#### ***Clinical data***

Clinical data were drawn from the medical records of the patients at the outpatient clinics. These included the type of disease, age at clinical diagnosis, disease duration and, for adolescents with diabetes, HbA_1c_ levels. Disease severity was evaluated by doctors in charge of the cases. Two questions (one about the level of the disease control and one about the patient’s current condition during the recent follow-up visit) each with four answer alternatives (“very poor” = 1, “poor” = 2, “good” = 3, and “very good” = 4) were asked in paper-pencil format. Physicians defined acceptable disease control based on the following determinants: (a) epilepsy – type of seizure, seizure frequency, antiepileptic medications and observed side effects, number of hospitalizations; (b) asthma – frequency of asthmatic episodes, medication side effects, number of hospitalizations; and (c) diabetes – level of metabolic control, number of hypoglycaemic episodes, disease complications, and number of hospitalizations. These scores were summed. Total score of ≥6 were designated “non-severe”, and scores of ≤5 coded “severe”. The score of 5 was selected for the severe disease status because it required at least one of the categories to be evaluated as “poor” or “very poor”.

#### ***Socioeconomic status***

Since no SES classification scheme was available for Russia, we used the mother’s/father’s education level, occupation level, and income level. For education, completed high school scored 0, college education and incomplete university education scored 1, and five or more years of university scored 2. For occupation, unskilled manual workers scored 0, skilled manual workers scored 1, and white-collar workers scored 2. For income level, there were three alternatives: low scored 0, average scored 1, and high income scored 2. The income level score were created reached by the grouping of data, dividing the study population income reports into 3 groups, representing each low, average, or high 33% of the data.

In order to analyse the relationship between HRQoL and socioeconomic variables we performed certain recoding manipulations. In two-parent families, the highest rating for parent occupation and education was used. The categories were then summed into the following classes, where 2 = high family SES (range 5-6), 1 = average SES (3-4), and 0 = low SES (0-2).

Family type – a parameter reflecting two- or single- parent household was included into the study analysis.

### Ethics

There was no ethical research committee in Arkhangelsk at the time of project planning and data collection. Informed consent was obtained from the head of the respective child outpatient clinic. All participants were informed that participation in the study was voluntary and confidential. Informed consent was obtained from all the participants (both mothers and adolescents). Participants were assured that outpatient clinic staff would not receive any private information from the questionnaires.

### Statistical analysis

All variables are presented as means with standard deviation. Cronbach’s alpha was used to determine the internal consistency reliability of the questionnaires. Fisher exact test was used to determine the relationship between socio-demographic characteristics of the study sample. Groups were compared using Student *t*-test and Mann–Whitney test. Individual domain and overall scores for the disease-specific QoL questionnaires were analysed as dependent variables in a linear regression analysis. Bivariate correlations were performed in order to exclude noncontributory variables. For the DQOLY, PAQLQ and QOLIE-AD-48 scores, we chose independent variables with moderate and high correlations (≥0.4). Thereby four variables – HbA_ic_ level, disease duration, age of onset and family type – were not included in the model. For all analyses, a two-tailed P < 0.05 denoted statistical significance.

## Results

### Descriptive clinical and demographic variables

There were no significant differences found in overall SES parameter between CPI groups.

According to medical records, the mean disease duration was as follows: diabetes 5 (±3) years, asthma 8 (±2.7) years, and epilepsy 6.6 (±3) years. The disease duration for adolescents with asthma was significantly longer than for those with diabetes (p < 0.001) or epilepsy (p < 0.01). The disease duration in the diabetes group was significantly (p < 0.05) shorter than for adolescents with epilepsy. The results of the disease severity evaluation made by doctors in charge of the cases are shown in Table 1. In the diabetes group the median HbA_ic_ level was 10.42% (interquartile range 12.1% - 7.3%), and the median number of daily insulin injections was five (interquartile range 6% - 5%). A combination of long and short-acting insulin was the most common treatment in diabetic patients.

### DQOLY

The internal consistency reliability was found to be satisfactory for all DQOLY subscales. Taking into consideration the different possible score ranges in each subscale of the instrument (17-85 for Satisfaction, 23-115 for Impact, 11-55 for Worries, and 51-255 for the Total DQOLY subscale), we transformed scores into a 0-100% scale to facilitate interpretation, where lower scores indicate better QoL. Adolescents with diabetes expressed most concerns regarding the impact of the disease and life satisfaction. 52% of adolescents estimated their overall health as “Fair”, 36% as “Good”, 8% as “Excellent”, and 4% as “Poor”. Girls showed significantly more Disease-Related Worries than boys (p < 0.05). None of the DQOLY scales were correlated with the level of metabolic control as measured by HbA_1c_ values, or with general self-rating of overall health. Means, standard deviations and Cronbach alpha coefficients for DQOLY are given in Table 2.

**Table 2:** Scale statistics for three diabetes QOL measures, total score, and Chronbach alpha coefficients

	**Satisfaction**	**Impact**	**Worries**	**Total**
Mean adolescents^1^	36.84 ± 11.40	50.36 ± 11.35	20.42 ± 7.02	107.6 ± 21.7
Girls (n = 22)	35.27 ± 9.88	47.59 ± 11.73	22.68^*^ ± 6.99	105.54 ± 23.56
Boys (n = 28)	38.07 ± 12.50	52.53 ± 10.75	18.64 ± 6.64	109.25 ± 20.44
Mean transformed	29.1 ± 16.7	29.7 ± 12.3	21.4 ± 15.9	80.3 ± 32.6
Cronbach alpha	0.88	0.82	0.79	0.88

^1^Percentile scale, lower level better QoL.

^*^p < 0.05 significant differences between girls and boys scores.

### PAQLQ

The internal consistency reliability, assessed with the Cronbach alpha coefficient, was satisfactory, with all subscales above the conventional standard of ≥0.70. The possible total score range was from 1 (maximum impairment) to 7 (no impairment); the sample range was from 3.35 to 7. The mean Overall score was 5.7, SD 0.92. The QoL scores in our study sample varied from the lowest score for the Activity domain to the highest on the Symptoms domain. There was a significant difference between girls’ and boys’ reports on PAQLQ, with girls scoring lower on the Symptom domain (p < 0.001) and Overall score (p < 0.01). Thus, girls reported more impairment in QoL compared to boys. Means, standard deviations, significant gender differences in PAQLQ subscale scores and Cronbach alpha coefficients are given in Table 3.

**Table 3:** Description of the adolescent’s total and domain scores in the PAQLQ, Chronbach alpha coefficients and gender differences

**X**	**Overall score**	**Activity**	**Symptom**	**Emotional**
Mean (SD)^1^				
Adolescents	5.7 (0.92)	5.2 (0.99)	5.9 (1.04)	5.8 (0.92)
Girls (n = 23)	5.3 (1.02)^**^	5.0 (0.85)	5.3 (1.22)^***^	5.6 (1.08)
Boys (n = 26)	6.0 (0.73)	5.6 (1.19)	6.3 (0.56)	6.0 (5.7, 6.6)
Cronbach alpha	0.94	0.72	0.89	0.85

^1^Higher score, better QoL.

^**^p < 0.01, ^***^p < 0.001 significant differences between girls and boys scores.

### QOLIE-AD-48

Internal consistency, assessed by Cronbach’s alpha coefficient, was found to be satisfactory (≥0.70) only in the Memory/concentration, Attitude, Stigma and Total subscales. The subscales Impact, Physical functioning, Social support, School behaviour, and Health perception were excluded from the analysis due to low internal consistency. Reliability analysis performed in the Brazilian study also revealed unsatisfactory results for three out of eight subscales, but Cronbach’s alpha coefficient for the total score was sufficient, as well as stability assessed by the Intraclass Correlation Coefficient [27]. The sample range for the subscales was from 0 to 100. Our subscale and total mean scores are similar to those of the original American-English version by J. A. Cramer [24], except for the Stigma subscale which is considerably lower in our study. Compared to boys, girls showed higher self-perceived QoL on the Memory/concentration subscale (p < 0.05) and for the Total score (p < 0.01), but lower on Attitude toward epilepsy (p < 0.05). Descriptive statistics and reliabilities of QOLIE-AD-48 subscales are shown in Table 4.

**Table 4:** Descriptive statistics and reliability of QOLIE-AD-48 subscales

**Subscale**^**1**^	**Mean score (SD)**	**Girls (29)**	**Boys (19)**	**Cronbach’s alpha**
Impact	74.48 (8.7)	77.15 (8.5)	70.54 (7.75)	0.41
Memory/concentration	69.9 (17.8)	74.31^*^(18.76)	63.28 (14.45)	0.89
Attitude	36.9 (21.4)	32.36 (23.32)	43.75^*^ (16.79)	0.89
Physical functioning	70.5 (14.2)	71.55 (11.88)	68.94 (17.44)	0.42
Stigma	59.8 (20.7)	64.48 (23.63)	52.92 (13.54)	0.81
Social support	91.4 (8.11)	88.57 (7.29)	95.72 (7.52)	0.68
School behavior	91.2 (10.5)	88.57 (11.93)	95.39 (6.19)	0.49
Health perception	60.4 (8.8)	62.64 (9.07)	57.01 (7.48)	0.18
Total	67.1 (7.1)	69.10^** ^(6.88)	64.13 (6.42)	0.74

^1^Higher score, better QoL.

^*^p < 0.05, ^**^p < 0.01 significant differences between girls and boys scores.

### The relationship between demographic and clinical variables and disease-specific QoL measurements

Linear regression analysis was performed for QoL predictors for each disease-specific QoL questionnaire (Table 5). HRQoL was best predicted by the independent variables for asthma and epilepsy groups with a maximum of 34% regarding the subscale Symptoms in asthma. Total PAQLQ and QOLIE-AD-48 reached 24% and DQOLY only 6%. Regarding single explanation variables, *disease severity* was most present in the diabetic group, *gender* in the epilepsy group, and *gender* and *SES* in asthma group. *Disease severity* was a significant predictor only in diabetes and asthma groups. For all three groups *gender* had an evident impact. *Age* was a significant predictor only in the epilepsy group.

**Table 5:** Multiple regression analysis with DQOLY, PAQLQ and QOLIE scales; the impact of demographic variables and disease severity

**Response variables**	**Explanatory variable (standardized regression coefficients (t-value))**				
	**Age**	**Gender**	**Disease severity**	**SES**	**Adjusted r**^**2**^
**DQOLY total**			0.29^*^ (2.11)		0.06
Impact				-0.30^*^ (2.16)	0.07
Worry		0.27^*^ (1.97)	0.31^*^(2.17)		0.10
Satisfaction			0.33^*^ (2.41)		0.09
**PAQLQ total**		-0.32^*^ (-2.37)		0.35^**^ (2.61)	0.24
Symptoms		-0.42^**^(-3.80)		0.36^**^ (2.89)	0.34
Activity			-0.36^**^ (-2.57)		0.11
Emotional				0.36^**^ (2.59)	0.11
**QOLIE total**	0.40^**^ (3.11)	0.35^**^ (2.72)			0.24
Memory		0.29^*^ (2.04)			0.06
Attitude	0.52^***^ (4.20)	-0.25^*^ (-2.06)			0.31
Stigma		0.28^*^ (1.98)			0.06

^*^p < 0.05, ^**^p < 0.01, ^***^p < 0.001.

The directions of associations were: less disease severity, higher SES, and older age were related to higher HRQoL. In the diabetes and asthma groups, girls expressed more concerns about their HRQoL than boys; the opposite was found in the group of adolescents with epilepsy in all but Attitude towards epilepsy subscale.

## Discussion

Health-related quality of life was studied in three cohorts of paediatric patients using disease-specific instruments, focusing on the self-perceptions of adolescents with diabetes, asthma or epilepsy. The outcome of the diabetes QoL scale (DQOLY) is quite reassuring. Transformed scores of three subscales indicated a relatively high level of HRQoL, although 56% of adolescents with diabetes evaluated their overall health in a negative direction. This discrepancy in our data would therefore suggest that the perception of HRQoL in adolescents with diabetes is influenced not only by health related parameters but by the complex of non-disease related determinants. The maintaining of optimal HRQoL in children with type 1 diabetes was stated by Wagner et al., reporting similar levels of HRQoL in diabetic participants as in their healthy peers [29]. In our sample, adolescents expressed less Disease-Related Worries, but lower Life Satisfaction compared to the original Ingersoll and Marrero study [18]. Adolescents with asthma rated their HRQoL toward the positive end of each specific scale. The overall score of PAQLQ calculated from our study sample reports was relatively high and similar to the results described in other studies [11,14]. Adolescents expressed most concerns in the Activity limitations domain. Asthma is a disease leading to a more restricted lifestyle; Williams [13] described adolescents’ perceptions of these limitations. The most significant considerations of patients with epilepsy were expressed in two subscales – Attitudes toward epilepsy and Stigma. The same kinds of conclusions were drawn by Wang and colleagues in validation of the Chinese version of QOLIE-AD [25]. Negative attitudes in society towards epilepsy can be the obvious and complex explanation of this finding, since stigma has an adverse impact on patients’ psychological wellbeing and QoL [30]. For many patients, stigma is a continuing social reality of their condition [31]. This may lead to adolescents incorporating CPI into their individual personal and social identities in diverse ways, which can affect how they choose to live with the illness [13] as well as how they perceive their HRQoL.

There are many aspects that comprise HRQoL for adolescents with a chronic disorder. However, they can be divided into two spheres: disease-related and non-disease-related factors. Disease-related factors include disease severity, age of onset, complications, sense of normality living with the disease, positive attitude toward the disease, and treatment. Non-disease-related factors include age, gender, SES, support of parents, social wellbeing and support [4]. The results of our analysis showed that four factors determined HRQoL in CPI adolescents – disease severity, gender, age, and SES.

Disease severity predicted the HRQoL outcome in the diabetic group and to some extent in the asthma group. The direction of the regression coefficient sign was evident – the higher was disease severity, the lower was HRQoL. Metabolic control expressed in HbA_1c_ is the most common indicator of disease severity in diabetic patients, and it has been shown to predict QoL in adolescents with diabetes [19]. We came to the same conclusion using a different severity measure, although there was no association between HbA_1c_ level and HRQoL in our study sample. However, the overall rather high HbA_1c_ levels in our sample could influence our analysis. Ingersoll and Marrero [18], who developed the DQOLY, showed a similar outcome. Our results for the asthma group correspond with a recent study by Alvim et al., where severity of clinical symptoms and morbidity were associated with HRQoL in adolescents with asthma [8]. These results should be interpreted with caution, because of different methods for disease severity evaluation. The proper spirometric procedure for asthma patients and a more structured disease-severity scoring in patients with epilepsy, as described by Austine et al. [32], could very well increase the correspondence between HRQoL scores and measures of severity. Unfortunately, however, it is not possible to find a common measure of severity appropriate for all disorders [9].

Gender predicted HRQoL in all three studied groups. The role of gender in the perception of HRQoL has been described in earlier research [10-12]. An explanation for the gender differences in HRQoL perception of CPI adolescents might lie in the characteristics of their conditions. In our sample, girls with diabetes showed more disease-related Worries than boys; and girls with asthma reported more Symptoms and overall lower HRQoL than boys. As suggested by Williams [13] in analysis of the same two disease groups, girls accept and express more problems and fears connected to their CPI. Boys by contrast attempt to maintain control over their disease by not letting it into their personal and social identities. We expected this to be even more pronounced in reports by boys with epilepsy, considering the relative degree of stigma attached to this disorder. However, boys with epilepsy in our sample demonstrated their HRQoL concerns. Bilgiç et al. found that adolescent boys expressed more emotional problems than girls, and suggested that in epilepsy male gender may be a risk factor for emotional symptoms [33]. Stigma is closely connected to gender and illness management. For boys, illness might be an isolating and threatening experience in a society where a boy is expected to be physically fit and tough [13]. Age as a determinant of QOLIE (the older the patient is the better the self-perceived QoL) accords with this latter observation given the difference in “puberty timing” in males and females [3], and might be explained by adjustment to chronic illness after the adolescent reaches puberty. Nevertheless, boys showing lower HRQoL contradicts earlier results obtained using the Brazilian versions of QOLIE-AD, where girls tended to have lower scores [27].

In our sample socioeconomic status predicted HRQoL in patients with asthma and diabetes. Our interpretation of the latter finding lies in the ground of family SES itself. It is constructed of three characteristics – parental education, occupation and income level. The first one is crucially important for the ability to explain to the child the importance of the compliance with the health regimens, positively motivating and developing the sense of normality resulting in the high HRQoL. It has been reported in Apter et al. study of the adult population [34] that cultural and socioeconomic factors influence the outcome of the disease, along with severity status. The impact of the income level in our study is disputable taking into consideration that all medical health care (in the hospitals or outpatient clinics), as well as medication expenses are covered by the state for all three studied groups. So in our work the accessibility to the convenient health care was controlled to some degree. Same degree of control by surveying patients of specialists was reported by Erickson et al. concluding that the household income was the most consistently associated factor with HRQoL for asthma paediatric patients and their caregivers [35].

### Limitations

The results of this study reflect the HRQoL of a limited population of adolescents due to the relatively small and homogeneous study sample. The convenience sampling technique was used in all studied groups but one, where adolescents with diabetes were true population representatives of their age in the city of Arkhangelsk. The cross-sectional design of the study precluded conclusions about the direction of association between predictors and HRQoL variables.

To reveal the issues of the target population groups, we chose to use disease-specific HRQoL instruments. It was therefore not possible to compare HRQoLs of different disease groups or to conduct a comparison with controls.

## Conclusions

The most reassuring finding of this study was that most of the adolescents with chronic illnesses estimated their HRQoL toward the positive end of the respective disease-specific scale. Gender was the only explanatory variable revealed in all three studied groups. It is of great importance to know how gender interacts differentially with chronic conditions such as diabetes, asthma and epilepsy. The linking of health disadvantages with gender has mostly focused on women, but there are studies indicating that hegemonic masculinities can also place the health of men at risk [36]. Our study suggests that future psychosocial interventions should focus on aspects of CPI impacting adolescents in gendered ways, also taking into account other factors that are disease specific in our sample (for diabetes, disease severity; for asthma, SES; and for epilepsy, age), affecting HRQoL.

For a complete understanding of HRQoL and its predictors in a CPI adolescent population, our results and observations require further analysis, including parents’ perspectives on the HRQoL of their adolescents, and dimensions of family functioning. Further research on CPI adolescent self-esteem, self-concept, as well as the exploration of adolescent’s social identity would help us to fulfill their needs and hopes of the health care professionals, create new educational programs based on an evaluation of each adolescent expectation.

## Abbreviations

HRQoL: Health related quality of life; QoL: Quality of life; CPI: Chronic physical illness; SES: Socio-economic status; DQOLY: The diabetes quality of life questionnaire for youths; PAQLQ: Pediatric asthma quality of life questionnaire; QOLIE-AD-48: Quality of life in epilepsy inventory for adolescents; HbA1c: Glycosylated hemoglobin.

## Competing interests

The authors declare that they have no competing interests.

## Authors’ contributions

AZ was responsible for the study design, data collection, data analysis, and drafting the manuscript. BH was the study supervisor, who made substantial contribution into the study design, analysis and interpretation of data, and revised the manuscript. Both authors read and approved the final manuscript.
